# Compensatory evolution of chromosomes and plasmids counteracts the plasmid fitness cost

**DOI:** 10.1002/ece3.70121

**Published:** 2024-08-20

**Authors:** Ziyi Liu, Qiuyun Zhao, Chenggang Xu, Houhui Song

**Affiliations:** ^1^ Key Laboratory of Applied Technology on Green‐Eco‐Healthy Animal Husbandry of Zhejiang Province, Zhejiang Provincial Engineering Laboratory for Animal Health Inspection & Internet Technology, Zhejiang International Science and Technology Cooperation Base for Veterinary Medicine and Health Management, China‐Australia Joint Laboratory for Animal Health Big Data Analytics College of Animal Science and Technology & College of Veterinary Medicine of Zhejiang A&F University Hangzhou China

**Keywords:** chromosome, compensatory evolution, fitness cost, plasmid

## Abstract

Plasmids incur a fitness cost that has the potential to restrict the dissemination of resistance in bacterial pathogens. However, bacteria can overcome this disadvantage by compensatory evolution to maintain their resistance. Compensatory evolution can occur via both chromosomes and plasmids, but there are a few reviews regarding this topic, and most of them focus on plasmids. In this review, we provide a comprehensive overview of the currently reported mechanisms underlying compensatory evolution on chromosomes and plasmids, elucidate key targets regulating plasmid fitness cost, and discuss future challenges in this field. We found that compensatory evolution on chromosomes primarily arises from mutations in transcriptional regulatory factors, whereas compensatory evolution of plasmids predominantly involves three pathways: plasmid copy number regulation, conjugation transfer efficiency, and expression of antimicrobial resistance (AMR) genes. Furthermore, the importance of reasonable selection of research subjects and effective integration of diverse advanced research methods is also emphasized in our future study on compensatory mechanisms. Overall, this review establishes a theoretical framework that aims to provide innovative ideas for minimizing the emergence and spread of AMR genes.

## Graphical abstract

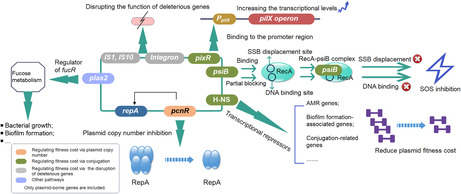

## 1 INTRODUCTION

Plasmids facilitate the horizontal transfer of genetic information among bacteria, allowing them to effectively adapt to diverse and intricate environmental circumstances. The transfer systems of many conjugative plasmids are rather promiscuous, enabling them to transfer DNA not only between similar species but also across unrelated species. Thus, plasmids are one of the main vehicles driving the genetic diversity in bacteria (Wiedenbeck & Cohan, 2011). However, for host bacteria, acquiring plasmids incurs fitness costs as well. For example, plasmids require additional resources for their replication, which can be a burden on the host cell. In addition, plasmids can disrupt cellular pathways and regulations, leading to metabolic burden on the host cell (Harrison et al., 2016). Therefore, to reduce the cost associated with these mobile genetic elements, bacteria and plasmids have various mechanisms, which can be categorized into six outcomes: (i) infectious transmission, which can be achieved by infectious spread, (ii) various host backgrounds can increase the probability of plasmid persistence, (iii) the epistasis effect generated by plasmid interactions can alter the fitness effects of plasmid acquisition, (iv) plasmids from more permissible hosts can maintain the presence of plasmids in non‐permissible hosts, which is called source–sink transmission, (v) piggybacking adaptations to novel environments can have pleiotropic effects on the cost of plasmid carriage, and (vi) compensatory evolution on the bacterial chromosome, plasmid, or both, ameliorating the plasmid fitness cost (Brockhurst & Harrison, 2022).

Compensatory evolution occurs when mutations arise that reduce or eliminate fitness costs, allowing the plasmid to persist in the bacterial population (Bouma & Lenski, 1988; Durao et al., 2018; Harrison et al., 2016; San Millan et al., 2014; Zhang, Fang, et al., 2022). Likewise, compensatory evolution is also thought to be one of the driving forces responsible for the extreme prevalence of specific plasmids and pathogenic bacterial lineages (Benz & Hall, 2023), as well as influencing the destiny of the bacterial population carrying multidrug resistance (MDR) plasmids (San Millan, 2018). Several studies have investigated compensatory evolutions in plasmids and bacteria (Rodriguez‐Beltran et al., 2021). For instance, pKP33, a large MDR plasmid, can achieve long‐term persistence in a naive *Escherichia coli* host through compensatory evolution (Porse et al., 2016). The presence of compensatory mutations can improve the overall permissiveness of MDR plasmids, and serve as an efficient and practical solution for the preservation of costly MDR plasmids. Despite a recent study proposing that plasmid‐located mutations are particularly effective in enhancing plasmid persistence (San Millan, 2018), the combinations of diverse host‐mediated mechanisms are responsible for plasmid fitness as well (Ares‐Arroyo et al., 2022). Therefore, the co‐evolution of plasmids and host bacteria is a complex and diverse phenomenon, necessitating a systematic review that summarizes the mechanisms underlying these compensatory evolutions.

This review provides a comprehensive overview of different compensatory evolutionary pathways based on previous studies, elucidating their respective effects. Specifically, the evolutionary mechanisms occurring on chromosomes and plasmids are listed separately in detail. Finally, we emphasize some promising key universal targets that have the potential to disrupt the persistence of MDR plasmids, along with the primary challenges in this field.

## 2 LITERATURE SEARCH STRATEGY

We searched the PubMed database using keywords “bacterial compensatory evolution” or “fitness cost” and “drug resistance.” The search results were limited to English‐language available online as of March 08, 2024, with no specified start date. A total of 1267 studies were retrieved. Based on these literature sources, we conducted a comprehensive screening and summarization of seven distinct compensatory evolutionary pathways on chromosomes as well as three types of compensatory evolutionary pathways specific to plasmids. In addition, we identified seven key sites associated with fitness costs on plasmids.

## 3 COMPENSATORY EVOLUTION ON CHROMOSOMES

Bacterial chromosomal genes contain essential genetic information for the functioning of various cellular processes. Compensatory evolution occurring on chromosomes predominantly involves global transcriptional regulators and varies by bacterial species. Due to the complexity of the mechanism underlying the regulation of plasmid fitness cost by chromosomal genes, most studies primarily focus on a phenotypical examination, without delving into the intricacies of the mechanism. The following are some examples.

### 3.1 Global regulatory system *gacA* / *gacS* and PFLU4242

The *gacA*/*gacS* two‐component system (TCS) exerts a positive regulatory role in enhancing the synthesis of extracellular proteins through the interaction with several small RNAs, which effectively suppress the activity of RNA‐binding proteins RsmE and RsmA, thereby alleviating post‐transcriptional repression (Sonnleitner & Haas, 2011). In laboratory conditions, compensatory mutations affecting the *gacA*/*gacS* have been found to rapidly arise in plasmid pQBR103‐carrying clones and were exclusive to plasmid carriers. The underlying mechanism may be due to mutations in this system that reduce the plasmid translational demand, as evidenced by the downregulation of approximately 17% of chromosomal and plasmid gene expression upon mutation of *gacA*/*gacS* (Hall et al., 2020; Harrison et al., 2015). The subsequent investigations revealed that these mutations not only impede plasmid loss within the population but also act as an obstacle to the integration of accessory traits into the bacterial chromosome (Harrison et al., 2016) and affect the coexistence of plasmid pQBR57 and pQBR103 in *Pseudomonas fluorescens* SBW25 (Carrilero et al., 2021). In addition, compensatory evolution has been shown to elicit the same impact in plant rhizosphere communities (Bird et al., 2023), indicating that the mutation of *gacA*/*gacS* may serve as an important strategy for *P. fluorescens* in facilitating the persistence of MDR plasmids within intricate communities. The *gacA*/*gacS* is widely distributed among plant pathogens (Chen et al., 2022). Therefore, elucidating its function in bacteria other than *P. fluorescens* may be crucial for preventing the dissemination of MDR plasmids among plants.

The chromosomal gene PFLU4242 is identified as a key determinant of plasmid persistence in *P. fluorescens* SBW25, as mutations in PFLU4242 were observed through compensatory evolution (Hall et al., 2020, 2021). The hypothesis suggests that this gene in *P. fluorescens* may have originated from a foreign source, as indicated by its absence in related strains but presence in distantly related genera, and its GC content differs from the overall genome (Hall et al., 2020). The domain DUF262, which belongs to the ParB superfamily, includes nucleases and partitioning systems as its principal components (Machnicka et al., 2015). Furthermore, the expression of PFLU4242 is induced by aforementioned TCS GacA/S. Mutation of GacA/S could potentially decrease plasmid cost by downregulating the expression of PFLU4242. Therefore, the mutation of GacA/S can regulate plasmid fitness cost by multiple pathways, whereas PFLU4242 plays a more proximal role in the regulation of plasmid cost, likely achieved by DNA breaks and subsequent activation of the SOS response (Hall et al., 2021).

### 3.2 Carbon catabolite repression (CCR) and aerobic respiration control (ArcAB) regulatory systems

CCR and ArcAB regulatory systems are important mechanisms in bacteria that regulate carbon metabolism and respiration, respectively (Brown et al., 2022; Shimizu, 2013). CCR ensures that the cell utilizes carbohydrates sequentially and relies on its preferred carbon source (Zhou et al., 2013), whereas ArcAB is a two‐component system associated with the expression of genes involved in aerobic respiration. The activation of ArcAB occurs in anaerobic environments, whereas it is repressed in aerobic conditions (Gaudu et al., 2003; Liu & De Wulf, 2004; Shimada et al., 2011). The mutations in the chromosomal CCR and ArcAB regulatory systems have pleiotropic effects that enhance the maintenance of two MDR plasmids, pG06‐VIM‐1 and pK71‐77‐1‐NDM, in clinical *E. coli* strains (Kloos et al., 2021). However, there is limited information available on the mechanisms of CCR and ArcAB regulating the plasmid persistence. Their role in regulating plasmid stability may be distinct. The CCR mutation is anticipated to mitigate the cost of pG06‐VIM‐1 by regulating intracellular cAMP levels, as the parameters of evolved strains and ancestral strains are significantly different. Meanwhile, different unique mutations in ArcAB appear to reduce plasmid cost by affecting bacterial transcriptional levels (Kloos et al., 2021). In contrast to the regulatory function of *gacA*/*gacS* mutation, which specifically regulates plasmid cost and is not observed in plasmid‐free evolved lineages, the adaptation in the CCR and ArcAB systems is not limited to plasmid‐carrying populations. The expansion of validation to a wider range of *E. coli* lineages and plasmid types will enhance our understanding of the pivotal role played by *E. coli* in maintaining and disseminating MDR plasmids (Pitout & Chen, 2023).

### 3.3 Global transcriptional regulator gene ferric uptake regulator (*fur*)

A SNP mutation (A53T) in *fur* gene, which could be considered a candidate target of alternative therapeutic interventions to slow down the spread of AR, has been verified to improve the persistence of a cryptic IncP‐1β in *Shewanella oneidensis* ATCC700550 (Stalder et al., 2017). The authors hypothesized that the presence of *fur* exerted a negative impact on plasmid persistence, while mutation might attenuate its regulatory effect. However, the hypothesis remains untested. Furthermore, the function of Fur in the biology of *Pectobacterium carotovorum* subsp. *brasiliense*, a significant potato pathogen, has been investigated as well. The *fur* mutant strain exhibited diminished virulence and fitness compared to the wild‐type strain, suggesting that the expression of *fur* is necessary for the bacterium to survive and thrive in its environment (Tanui et al., 2017). The Fur protein is widely distributed in most prokaryotes (Pasqua et al., 2017), and functions as a transcriptional repressor protein that responds to iron levels (Thompson et al., 2002). It has been shown to impact the biofilm formation ability of bacteria (Latorre et al., 2018). Biofilms facilitate plasmid stability and may potentially enhance the host range of horizontally transferred mobile elements (Roder et al., 2021). Meanwhile, bacteria within biofilms generally exhibit reduced metabolic activity compared to their planktonic counterparts, thereby mitigating the likelihood of plasmid loss (Madsen et al., 2012). Therefore, exploring the impact of Fur on bacterial biofilm formation and revealing its role in improving plasmid stability can serve as an alternative direction for future investigations.

### 3.4 Helicases

The helicases, a class of enzymes, act as a crucial part of various DNA and RNA metabolic processes. They can be categorized into two classes: replicative helicases responsible for replication, and accessory helicases involved in DNA repair, recombination, transcription, and resolution of replication–transcription conflicts (Merrikh et al., 2012, 2015). Currently, at least four researches have suggested that host‐encoded helicases are able to affect plasmid cost and persistence (Loftie‐Eaton et al., 2016, 2017; San Millan et al., 2014; Sota et al., 2010). Among them, the loss of helicase function or its binding domain through experimental evolution can reduce the fitness cost of plasmid pNUK73 and IncP‐1β plasmid pBP136 (San Millan et al., 2014; Sota et al., 2010), whereas others believed that the high transcriptional level of helicase is beneficial for the persistence of plasmid RP4 (Loftie‐Eaton et al., 2017). The mode of helicase action may potentially vary depending on the specific plasmid type and host bacteria. Despite the underlying mechanism is still unexplored, several studies have postulated that helicase may influence plasmid cost and persistence by modulating plasmid copy number or replication efficiency (Loftie‐Eaton et al., 2017; Yano et al., 2016).

### 3.5 RNA polymerase (RNAP)

The bacterial RNAPs primarily consist of *rpoA*, *rpoB*, *rpoC*, *rpoD*, and *rpoZ* genes encoding the core α, β, β′, σ^70^, and ω subunits, respectively (Borukhov & Nudler, 2003). Mutations in these RNAP genes have the potential to cause pleiotropic effects on bacterial phenotypes (Jin & Gross, 1988; Zhou & Jin, 1998), and may confer various selective advantages (Murphy & Cashel, 2003). Mutations in these genes following adaptive evolution play a predominant role as driving forces mediating the compensatory evolutionary process. For example, the *rpoC* gene in *E. coli* MG1655 was found to undergo specific small deletions through evolution in minimal nutrients media, which had a profound impact on the bacteria at physiological, molecular, and global transcription levels, suggesting that mutations in RNAP can potentially reprogram the kinetic parameters for optimal growth in new environments (Conrad et al., 2010). While the existing research does not directly address *rpoC* mutations in relation to plasmid fitness costs, the effects of *rpoC* mutation have been shown to reduce the plasmid fitness cost through growth conditions and environmental factors (Applebee et al., 2008; Zhang, Mao, et al., 2022). For example, an *rpoC* mutation (a 27 bp deletion) increased the metabolic efficiency of *E. coli* MG1655 to gain growth advantage, providing a greater fitness benefit (Cheng et al., 2014). In addition to *rpoC*, non‐synonymous SNPs of *rpoB* gene after the initial *rpoB* mutation that results in rifampin resistance have been reported to improve plasmid stability. However, unlike previous studies, the authors propose that this process is mediated by epistatic effects between helicase and *rpoB* mutations, as *rpoB* mutations alone do not appear to contribute to the improvement of plasmid stability (Loftie‐Eaton et al., 2017). The realization of genetic information can be influenced at various stages by mutations in RNAP, including promoter recognition, RNA synthesis, and protein synthesis on mRNA (Yarulin & Gorlenko, 1985). As a consequence, the effect of RNAP mutations can differ based on the particular mutation and its contextual presence. For instance, mutations in RNA polymerase genes can either enhance or diminish the termination of transcription by the *rho* factor at specific terminators in *E. coli* (Yarulin & Gorlenko, 1985). Additionally, the CRE pocket is an enzyme responsible for transcribing DNA to RNA. Its mutations in bacterial RNA polymerase have a broad impact on various stages of transcription, encompassing elongation and intrinsic termination (Petushkov et al., 2015). Plasmid stability is significantly affected by conflicts between transcription and replication processes. Transcriptional modifications can help reduce these conflicts, leading to more stable plasmid inheritance (Wein et al., 2019; Yang, Liu, et al., 2023). Thus, although most of the above studies have little relevance to plasmid fitness cost, RNAP mutations can be considered an efficient solution to reduce plasmid fitness cost.

### 3.6 Outer membrane porin

The mutation in outer membrane porin ompF, apart from global transcriptional regulators, has the ability to decrease the cost of horizontally acquired tetracycline resistance during the early stage, thereby exerting a significant phenotypic impact on both tetracycline resistance and growth (Bottery et al., 2019). This finding is also supported by our previous investigation, which revealed the presence of SNPs in OMPs through compensatory evolution of a cointegrate plasmid in *K. pneumoniae* YZ6, implying that the mutations of OMPs were likely to promote the plasmid survival (Liu et al., 2021). For host bacteria, the loss of OMP function leads to a reduction in fitness and impaired growth. However, it can be rapidly compensated via two pathways: (i) the *pho* regulon can be activated by mutations in *phoR* and *pstS*, leading to the activation of a group of genes involved in phosphate metabolism. Activation of the *pho* regulon facilitates bacterial acquisition of phosphate from the environment, which is indispensable for growth and survival. (ii) Perturbing Hfp‐dependent sRNA regulation through mutations in *chiX* and *hfq*, leading to the induction of *chiP* expression. This pathway can help the bacteria to degrade and recycle OMPs, thereby compensating for the loss of OMP function (Knopp & Andersson, 2015). Therefore, OMP mutations may reduce the plasmid fitness cost by modulating a cascade of downstream pathway alterations, such as phosphate metabolism.

### 3.7 Oxidative or osmotic stress‐associated genes

In *Salmonella typhimurium* ATCC14028, mutations in oxidative stress‐associated gene *ahpC*, *ybgS*, as well as osmotic stress‐associated gene *osmY*, have been shown to drive the plasmid–host coevolutionary processes. The study found that knockout mutants have a higher competitive advantage than wild‐type strains, but the mechanism remains to be elucidated (Zhang, Fang, et al., 2022). *ahpC* and *ybgS* are genes involved in antioxidant activity (Sherman et al., 1999), whereas *osmY* is linked to osmatic shock and bacterial state (Yan et al., 2019; Zheng et al., 2015). Mutations in these genes may change the bacterial physiological state under environmental stress, thereby overcoming the plasmid fitness cost. In *E. coli*, the mutations in genes associated with oxidative stress appear to be linked to the mitigation of specific plasmid–host genetic conflicts and the reduction of fitness costs caused by a *bla*_NDM_‐positive IncX3 plasmid (Li et al., 2023).

### 3.8 Changes in gene expression levels related to flagellar synthesis

Theoretically, the changes in expression level of flagellar synthesis‐associated genes can directly or indirectly contribute to bacterial fitness. Flagella play an indispensable role in bacterial biofilm formation (Pratt & Kolter, 1998), which allows bacteria to thrive in various physiological conditions and promotes plasmid survival (Chew & Yang, 2016; Roder et al., 2021). In addition, the wild‐type strain of uropathogenic *E. coli* (UPEC) demonstrated superior competitiveness in the urinary tract compared to its flagella‐deficient counterpart, suggesting that flagella directly contributes to bacterial fitness. (Lane et al., 2005). Through compensatory evolution, alterations in gene expression levels associated with flagellar synthesis are frequently observed instead of mutations (Zhang, Fang, et al., 2022; Zhang, Mao, et al., 2022), and are usually accompanied by mutations in their upstream transcriptional regulators. For instance, in our previous study, it was demonstrated that mutation of *sspA* can alleviate its transcriptional inhibition and promote flagellar synthesis in evolved strains, thereby reducing the plasmid fitness cost (Liu et al., 2024). The presence of similar compensatory evolutionary patterns in clinical settings holds greater significance compared to laboratory settings. Because the enhanced flagellar synthesis ability in clinical pathogens could potentially pose greater threat to public health and complicate therapy due to their intricate association with bacterial virulence, motility, and plasmid persistence (Soutourina & Bertin, 2003) (Table 1).

**TABLE 1:** Summary of genes mediating compensatory evolution on bacterial chromosomes.

Genes	Function	Physiological activities	Speculative mechanism	Reported plasmids and species	References
*gacA*/*gacS*	Global transcriptional correlation	Quorum sensing, secondary metabolism, biofilm formation, and motility	Mutation of *gacA*/*gacS* may reduce the gene translation demand	Plasmid pQBW103 and pQBW58 in *P. fluorescens*	Hall et al. (2020), Harrison et al. (2015), Carrilero et al. (2021), Bird et al. (2023)
*CCR* and *ArcAB*	Global transcriptional correlation	*CCR*: carbon metabolism *ArcAB*: respiration	CCR: regulating intracellular cAMP levels ArcAB: affecting bacterial transcriptional level	Plasmid pG06‐VIM‐1 and pK71‐77‐1‐NDM in *Escherichia coli*	Kloos et al. (2021)
*fur*	Global transcriptional correlation	Metal ion uptake and metal homeostasis	Mutation of *fur* may attenuate its regulatory effect	IncP‐1β plasmid in *Shewanella oneidensis*; *Pectobacterium carotoyorum* subsp. *brasiliense*	Stalder et al. (2017), Tanui et al. (2017)
Helicases	Nucleic acid metabolism	DNA replication, repair, recombination, transcription, ribosome biogenesis, RNA processing, translation, and decay	Mutation of helicase‐related genes may modulate plasmid copy number or replication efficiency	Plasmid pNUK73 in *P. aeruginosa*; Plasmid pBP136 in *S. oneidenesis* Plasmid pS0506 in *Pseudomonas moraviensis*	Loftie‐Eaton et al. (2016, 2017), San Millan et al. (2014), Sota et al. (2010)
RNA polymerase	Transcriptional correlation	Transcribe RNA molecules using DNA as a temple	The effects of mutations in RNAP can vary depending on the specific mutation and its contextual occurrence	*E. coli*; *Mycobacterium tuberculosis*	Applebee et al. (2008), Zhang, Mao, et al. (2022)
PFLU4242	Unknown	Unidentified	A key mediator in regulating the plasmid fitness cost	Plasmid pQBW103 and pQBW58 in *P. fluorescens*	Hall et al. (2020, 2021)
Outer membrane porin	Bacterial transport system	Cellular permeability and antibiotic resistance	Mutations of alternative porins bypassed the need for functional other porins	Plasmid RK2 in *E. coli*; A fusion plasmid in *Klebsiella pneumoniae*	Bottery et al. (2019), Liu et al. (2021), Knopp and Andersson (2015)
Oxidative or osmotic stress‐associated genes	Stress correlation	Oxidative stress genes: elimination of ROS, cytoprotective functions, regulation of secondary metabolism Osmotic stress genes: regulation of the cell cycle, induction of gene expression, activation of transcription factors	Mutations of these genes may change the bacterial physiological state under environmental stress	Plasmid pJXP9 in *S. typhimurium*; A IncX3 plasmid in *E. coli*	Zhang, Fang, et al. (2022), Li et al. (2023)

## 4 COMPENSATORY EVOLUTION ON PLASMIDS

Different phases of plasmid biology incur fitness costs, including plasmid reception, integration, replication, conjugation, gene expression, and the interactions between mobile genetic elements (San Millan & MacLean, 2017). This section aims to discuss three main compensatory evolution patterns on plasmids and several identified targets for regulating the plasmid fitness cost.

### 4.1 Deletion of various fragments on plasmids

Compensatory evolution can occur not only on chromosomes but also on plasmids, with plasmid mutations having a more profound impact than chromosome mutations due to their ability to be inherited both vertically and horizontally. Therefore, plasmid‐located compensatory evolution exhibits superior efficacy in enhancing plasmid persistence (Alonso‐Del Valle et al., 2021), even when its impact is comparatively smaller than that of chromosomal compensation as indicated by mathematical models and simulations (Zwanzig et al., 2019). For large MDR plasmids, the high abundance of insertion sequences shapes their high plasticity, thereby conferring them remarkable adaptability allows for rapid host adaptation, which is often achieved through the elimination of costly fragments (Porse et al., 2016). Compensatory evolution involves the deletion of fragments is a common evolutionary strategy for plasmids. After this process, it is often accompanied by positive effects such as a reduction in plasmid fitness cost and an enhancement in plasmid stability. However, there exists a trade‐off as the physiological function of plasmids themselves may also be affected, leading to reduced conjugation frequency and antimicrobial resistance levels (Dorado‐Morales et al., 2021; Porse et al., 2016; Zhang, Fang, et al., 2022).

The occurrence of fragment deletion is frequent in both plasmid MDR region and conjugation transfer region, which may largely be attributed to the energetically expensive expression of these genes, including the conjugation genes (Zahrl et al., 2006) and some AMR genes (e.g., mobile colistin resistance gene *mcr‐1* (Yang et al., 2017), extended‐spectrum β‐lactamase gene *bla*_CTX‐M‐15_, and the tetracycline resistance determinants *tetAR* (Rajer & Sandegren, 2022)). The absence of AMR genes has minimal impact on the physiological function of plasmid, whereas the deletion of conjugation transfer genes exerts a more significant effect. The rearrangement of the conjugation transfer region may serve as the most effective compensatory mechanism to mitigate the fitness cost incurred by bacterial hosts during conjugation. Nevertheless, excessive manipulation of the plasmid conjugation transfer system could potentially disrupt its horizontal transfer ability, thereby impacting the epidemic distribution of plasmids. The MDR IncHI2 plasmid, pJXP9, undergoes a deletion event wherein both the MDR regions and conjugation transfer region I are discarded compensating for the fitness cost associated with plasmid carriage. While this deletion event enhances plasmid maintenance and vertical transmission capability, it compromises the plasmid horizontal transmission capacity and bacterial physiology (Zhang, Fang, et al., 2022). Likewise, in our previous research, we observed that a cointegrate plasmid pL53T underwent compensatory evolution resulting in the deletion of a large‐scale sequence. Although the fitness cost of host bacteria caused by the evolved plasmid is minimal, its ability to undergo horizontal transfer is significantly hindered due to the lack of the *psiB* gene (Liu et al., 2024).

Besides the laboratory condition, plasmid fragment deletions have been observed in clinical settings as well. Alvaro San Millan and his colleagues monitored the evolution of the clinically relevant plasmid pOXA‐48 in the intestinal tract of hospitalized patients and found that a large ~13.5 kb fragment deletion resulted in the loss of conjugation ability in host bacteria (DelaFuente et al., 2022). This suggests that such compensatory evolution patterns are common in both laboratory and clinical settings.

### 4.2 Acquisition of additional fragments on plasmids

In addition to deleting some fragments, acquiring foreign fragments is an alternative strategy for the plasmid fitness improvement, particularly resolvases and TA systems (Loftie‐Eaton et al., 2016; Stalder et al., 2017). TA systems are recognized as efficacious mechanisms for preventing plasmid loss in bacterial populations (Goeders & Van Melderen, 2014). The acquisition of a TA system by newly introduced plasmid consequently promotes their vertical transmission through the inhibition of cell growth in plasmid‐deficient cells (Stalder et al., 2017). Resolvases are recognized for their ability to resolve cointegrates, including plasmid multimers that frequently arise post‐replication (Field & Summers, 2011). The synchronized expression of the TA system and the resolvase may thus facilitate temporary inhibition of cellular division and resolve the multimers, thereby favoring plasmid persistence (Loftie‐Eaton et al., 2016). The occurrence of plasmids acquiring foreign fragments is comparatively less frequent than the deletion of fragments, potentially attributed to the additional metabolic burden imposed on host bacteria by expressing foreign genetic elements.

### 4.3 Plasmid‐borne mutations

The mutation of the plasmid itself can also facilitate the compensatory evolutionary process, a phenomenon commonly observed in genes associated with plasmid replication and conjugation (Ares‐Arroyo et al., 2022; Metzger et al., 2022; Porse et al., 2016; Stalder et al., 2017; Yang, Wu, et al., 2023). Interestingly, the mechanisms are independent between these mutations, despite both leading to plasmid persistence. Mutations associated with DNA replication are likely to compensate for the plasmid fitness cost (e.g., regulate the plasmid copy number), thus improving its plasmid persistence (San Millan et al., 2015; Sota et al., 2010; Yano et al., 2016). By contrast, mutations in conjugation‐related genes are more inclined to alter horizontal transfer ability to reduce plasmid fitness cost (Yang, Wu, et al., 2023), which highlights that plasmid usually displays a tradeoff between conjugation rate and fitness (Heuer et al., 2007). However, it is noteworthy that plasmids exhibit instability even in the presence of compensatory evolution, a phenomenon commonly observed when two functionally redundant plasmids are subjected to positive selection conditions (Carrilero et al., 2021). Meanwhile, the stability improvement of plasmids without compensatory evolution can also occur through the epistatic effect resulting from their interaction (Gama et al., 2020).

In addition to plasmid housekeeping genes, certain plasmid‐encoded accessory genes that mediate bacterial adaptation to the environment are considered a significant potential source of fitness cost due to specific genetic conflicts between chromosomal and plasmid genes. For example, the overexpression of *mcr‐1* is deleterious for host bacteria. However, to balance the fitness cost and *mcr‐1* expression, bacteria evolved a fine‐tuned regulatory evolution that optimized the expression of *mcr‐1*, resulting in a lower fitness cost and enhanced colistin resistance (Ogunlana et al., 2023). Furthermore, the plasmid‐encoded phage gene *relA*_*P1*_ interacts antagonistically with *bla*_TEM‐116_, but the deletion or point mutations in *relA*_*P1*_ can effectively mitigate the cost associated with *bla*_TEM‐116_, which highlights the influence of plasmid accessory genes on the evolution dynamics of AMR gene (Lai & Cooper, 2024). In general, the occurrence of compensatory mutations in accessory genes is less frequent than in housekeeping genes. Even if mutated, some of these genes do not exhibit an obvious association with improved fitness costs. Owing to the instability of plasmid accessory genes, targeting housekeeping genes may be more suitable for preventing plasmid transmission.

## 5 IDENTIFIED KEY TARGETS ASSOCIATED WITH THE PLASMID FITNESS COST

### 5.1 A ProQ/FinO family protein

The PcnR protein, belonging to the ProQ/FinO family, is identified as a plasmid copy number repressor and is located on IncI2 plasmids. It regulates the level of *mcr‐1* gene expression and bacterial viability by suppressing plasmid replication (Yang et al., 2021). The plasmid‐encoded ProQ/FinO system has been exclusively associated with the regulation of conjugation in IncF plasmids (Olejniczak & Storz, 2017). However, the identification of *pcnR* has expanded our knowledge of the function attributes of ProQ/FinO family proteins. Considering that IncI2 plasmids are widely recognized as highly successful vectors for disseminating various antimicrobial resistance (AMR) genes on a global scale, the identification of *pcnR* gene function can provide an important reference for the precise prevention and control of the transmission of IncI2 plasmids (Quan et al., 2017). It serves as not only one of the limited replication control components but also a potential target for controlling the dissemination of AMR genes based on its mechanism, which involves binding to the initial stem–loop structure of repR mRNA to regulate plasmid copy number.

### 5.2 *PixR* , *psiB* , and H‐NS protein

The increase in conjugation rate is an alternative strategy to counterbalance the plasmid fitness cost. PixR has been found to play a crucial role in the regulation of IncX4 plasmid transfer by binding to the promoters of 13 key transfer genes, which leads to an increase in their transcriptional activity (Yi et al., 2022). The existence of *pixR* is seen as an alternative account for the worldwide occurrence of *mcr‐1*‐carrying plasmids, as IncX4 plasmids are identified as the second most widespread epidemic carriers of *mcr‐1* following IncI2 plasmid (Liu, Zhou, et al., 2020).

The *psiB* gene, usually detected in IncF plasmids, is associated with the suppression of SOS response induced by conjugation (Petrova et al., 2009), thereby significantly ensuring the transfer efficiency of plasmids. The influence of plasmids on their prevalence has been demonstrated in both laboratory and natural environments. A recent study revealed that the absence of *psiB* in plasmid pED208 resulted in a significantly stronger SOS response compared to its counterpart, potentially hindering the horizontal transfer of this plasmid (Al Mamun et al., 2021). Furthermore, our research revealed that the deficiency of *psiB* gene is responsible for the impaired horizontal transferability of a cointegrate plasmid through compensatory evolution (Liu et al., 2024). In a study conducted in natural environments, the epidemiological characteristics of two plasmids, namely pKSR100 and pAPR100, were investigated. These plasmids were found to circulate within the same network but exhibited distinct properties. Plasmid pKSR100 demonstrated a higher ability for conjugation compared to the less prevalent plasmid pAPR100. However, it showed a reduced SOS response when exposed to antimicrobials. Through comparative genomic analysis, it was discovered that pKSR100 contained a gene cluster comprising five genes, including the *psiB* gene, which could potentially account for the difference in prevalence observed between these two plasmids (Malaka De Silva et al., 2022).

The H‐NS protein, referred to as a histone‐like nucleoid structuring protein, acts as a transcriptional inhibitor that represses the expression of acquired genes (Dorman, 2007; San Millan & MacLean, 2017). H‐NS thus plays an essential role in modulating the plasmid fitness cost. The absence of H‐NS‐like protein in IncX3 plasmids results in a 2.5‐fold increase in their transfer frequency, underscoring the crucial role played by this protein in the dissemination of IncX3 plasmids (Liu, Shui, et al., 2020). Additionally, the H‐NS protein may exert a regulatory influence on the fitness of IncX1 plasmids carrying *tet*(X4) by suppressing the expression of the *tet*(X4) gene (Cai et al., 2021). Taken together, H‐NS exhibits versatility in modulating various plasmid fitness costs.

### 5.3 Insertion sequences and integrons

The pivotal role of mobile genetic elements in facilitating the dissemination of AMR genes has been consistently emphasized, yet their contribution to mediating compensatory evolution of bacteria has been underestimated. Recently, both insertion sequences and integron have been found to lead to rapid plasmid compensatory evolution. For insertion sequences, IS*1* and IS*10* can decrease the plasmid fitness cost by disrupting the function of a deleterious gene on plasmid (Wedel et al., 2023). For integrons, they can facilitate plasmid adaptation by inducing off‐target inversions that disrupt conjugative genes (Souque et al., 2023). Therefore, the involvement of insertion sequences and integrons is indispensable in the development of AMR genes, both from a transmission and evolutionary perspective.

### 5.4 A novel intragenic region of small RNA *plas2*

**FIGURE 1:**
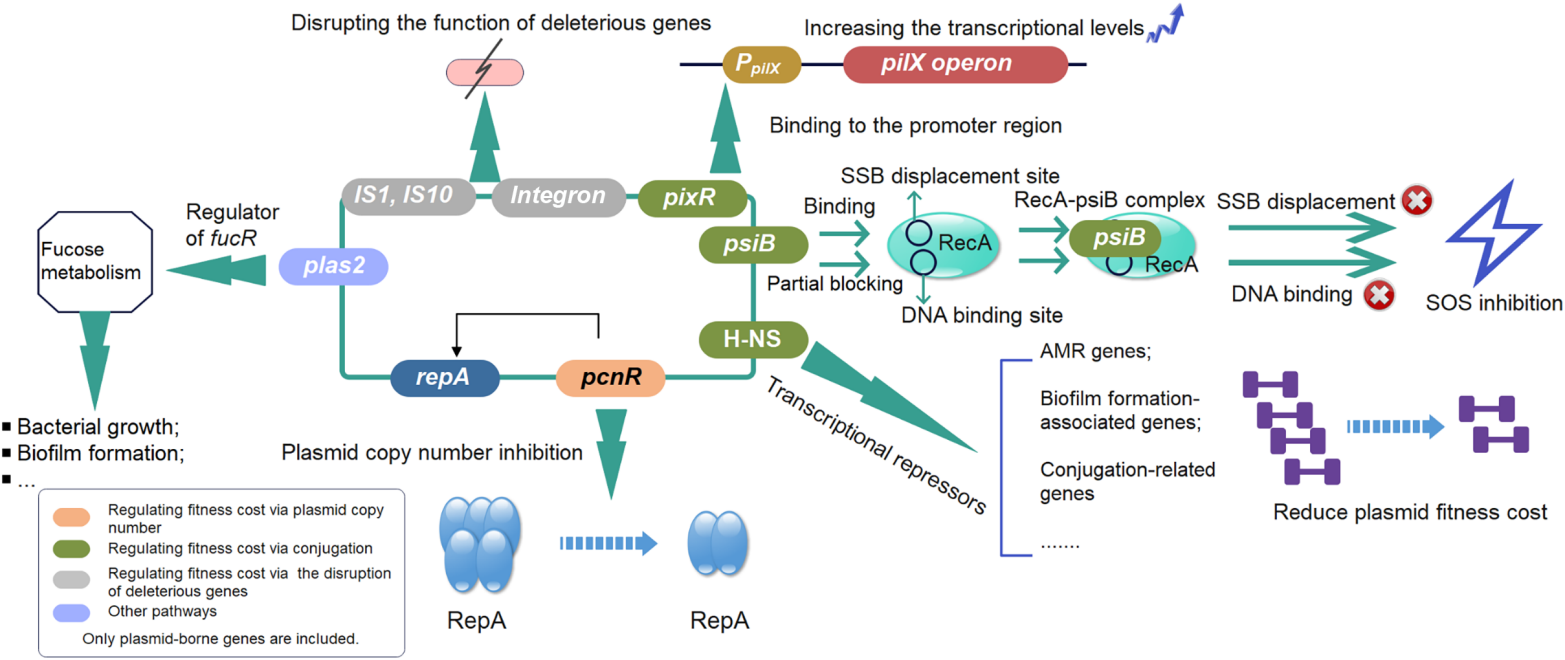
Schematic diagram of several plasmid‐borne genes associated with the plasmid fitness cost. The figure represents a hypothetical plasmid and the details in the figure have been drawn from studies on several different plasmids and study systems. Genes in the blue background represent replication gene *repA*, and the *pcnR* gene can control the plasmid copy number by binding to the *repA* gene. The genes *pixR*, *psiB*, and *hns* in the green background have all been shown to regulate plasmid fitness cost by impacting plasmid conjugation. The gray background represents the insertion sequences and integrons, which can regulate plasmid fitness by disrupting the function of deleterious genes. The purple background represents the small RNA *plas2* located on IncX3 plasmids, which can regulate bacterial growth and biofilm formation by fucose metabolism.

The *bla*_NDM_‐bearing IncX3 plasmid pNDM‐HN380 contains a novel intragenic region small RNA, known as *plas2*, which acts as a regulator of *fucR* gene involved in the fucose metabolism (Huang et al., 2020). In this way, *plas2* can regulate biofilm formation and flagella synthesis in bacteria that carry the plasmid. The successful identification of *plas2* can provide valuable insights into the underlying factors contributing to the global distribution of *bla*_NDM_‐bearing IncX3 plasmids. Furthermore, it offers novel perspectives on the potential application of plasmid‐transcribed sRNA as a strategy against multidrug‐resistant pathogens (Figure 1).

## 6 OUTLOOKS AND CHALLENGES

The fitness effects of plasmids have attracted increasing interest in recent years, and researchers are now beginning to identify some of the fundamental principles underlying these effects (San Millan & MacLean, 2017). Meanwhile, another challenge faced by researchers is that compensatory evolution perfectly circumvents the constraints of plasmid fitness cost, leading to the ubiquitous prevalence of MDR plasmids. These evolutionary pathways are mediated by chromosomes, plasmids, or both. The key to controlling the dissemination of MDR plasmids lies in elucidating the underlying molecular mechanisms governing plasmid evolution within host bacteria, either through laboratory evolutionary experiments or by studying well‐adapted host–plasmid combinations. Therefore, the first challenge is whether it is more promising to obtain a key target from laboratory evolutionary experiments or well‐adapted natural host–plasmid combinations. The limitation of laboratory evolutionary experiments is their confinement to controlled laboratory conditions, and most are engineered bacteria, thereby potentially hindering the reproducibility of similar outcomes in vivo or natural environments and resulting in some erroneous predictions, for example, plasmid‐associated fitness costs in the laboratory *E. coli* strain J53 tend to be much higher than in the wild‐type bacteria (DelaFuente et al., 2022). Conversely, the advantages of identifying key targets from specific bacteria–plasmid combinations found in nature are twofold. Firstly, the experimental subjects have undergone extensive natural selection, resulting in enhanced accuracy of predictions and broader applications. Secondly, the targets identified by the strategy typically involve general regulatory mechanisms, such as the plasmid copy regulator and conjugation rate. However, triggering the compensatory evolutionary mechanism under laboratory conditions may require harsh conditions like antibiotic selective pressure or nutritional conditions. Therefore, the investigation of multiple plasmids that facilitate the dissemination of crucial resistance genes as targets, aiming to elucidate the underlying molecular mechanisms governing their extensive circulation, is believed to be a promising research avenue in the future (Yang et al., 2021; Yi et al., 2022).

In the research field, it is essential to consider several advanced research tools and methods. Firstly, the foundation for compensation determination is established by conducting genomic and transcriptomic analyses in most relevant studies (Kawano et al., 2020; Yang, Liu, et al., 2023). However, a limited number of studies also incorporate other omics analyses (Ares‐Arroyo et al., 2022; San Millan et al., 2018).

While genomic and transcriptomic analyses offer valuable insights into genetic variations and gene expression changes, they may not fully capture the complex interplay between different biological layers involved in the evolutionary processes. By integrating multi‐omics datasets, researchers can gain a more comprehensive understanding of the molecular networks, regulatory mechanisms, and functional consequences associated with compensatory evolution. This integrative approach can uncover novel relationships and interactions between different biological components, enabling a systems‐level perspective on the evolutionary process (Roehrig et al., 2024). Moreover, population sequencing is being utilized to investigate alterations in the relative abundance of genes across generations (Wang et al., 2023; Zhang, Fang, et al., 2022). In comparison to monoclonal sequencing, this approach captures the dynamic changes in gene abundance, but it fails to capture the linkage between plasmid and chromosomal mutations at the single‐cell level. This is a critical shortcoming, especially for multicopy plasmids, where it is impossible to know which plasmid mutations co‐occur with specific chromosomal mutations within the same cell (Wang et al., 2021). Secondly, the utilization of mathematical models is a promising strategy to investigate the dynamics of plasmid evolution (Loftie‐Eaton et al., 2016, 2017; Prensky et al., 2021; San Millan et al., 2014; Yang, Wu, et al., 2023). It generally enables the provision of predictive outcomes, thus employing in silico analysis can enhance the persuasiveness of these results and conclusions. Thirdly, the current prevailing methods for phenotypic tests related to bacterial fitness, such as competition assays, are primarily based on traditional colony counting techniques. However, in recent years, some emerging measurement methods have become increasingly popular. The use of fluorescently labeled co‐cultures is a typical example that allows for precise measurement of selection dynamics in potential competition changes because it has the property of real‐time measurement compared to traditional endpoint measurement methods (Kehila & Tokuriki, 2024).

In conclusion, this review provides a comprehensive overview of the current molecular mechanisms driving compensatory evolution in host bacteria. Our objective is to establish a theoretical framework that can contribute to the development of innovative antimicrobial strategies aimed at minimizing the emergence of AMR. We found the targets of bacterial fitness cost improvement via chromosomal mutation are primarily global transcriptional regulators, whereas the plasmid evolution mechanisms are involved in the regulation of plasmid copy number and the control of expression for costly AMR genes or conjugative genes. Furthermore, challenges in this field including selecting appropriate plasmid–host bacteria, integrating various advanced techniques and methods, as well as their subsequent application deserve our attention.

## AUTHOR CONTRIBUTIONS

**Ziyi Liu:** Visualization (lead); writing – original draft (lead). **Qiuyun Zhao:** Data curation (equal). **Chenggang Xu:** Funding acquisition (equal); supervision (equal). **Houhui Song:** Funding acquisition (lead); supervision (lead).

## FUNDING INFORMATION

This work was supported by the National Natural Science Foundation of China (Grant number 32170053) and the Science Development Foundation of Zhejiang A&F University (Grant number 2024LFR030).

## CONFLICT OF INTEREST STATEMENT

The authors declare that they have no conflict of interest.

## Data Availability

No new data are included in this review. All previously published data have been made available through links in the original publications.
